# Shifts in acute respiratory pathogen epidemiology: an 8-year surveillance study in Hangzhou, China (2018–2025)

**DOI:** 10.3389/fpubh.2026.1907370

**Published:** 2026-09-30

**Authors:** Yi Wang, Liangliang Huo, Jing Wang, Xinye Jin, Jiayun Wang, Longfang Jiang

**Affiliations:** 1 Infectious Disease Control Department, Hangzhou Center for Disease Control and Prevention (Hangzhou Health Supervision Institute), Hangzhou, China; 2 Xiaoshan District Center for Disease Control and Prevention (Xiaoshan District Health Supervision Institute), Hangzhou, China

**Keywords:** Acute Respiratory Tract Infection, epidemiological characteristics, Hangzhou, influenza virus, *Mycoplasma pneumoniae*, pathogen spectrum

## Abstract

**Objectives:**

To analyze the epidemiological characteristics of six common respiratory pathogens in cases of Acute Respiratory Tract Infection (ARTI) in Hangzhou from 2018 to 2025, to provide a basis for clinical diagnosis and treatment, as well as public health prevention and control.

**Methods:**

A multicenter retrospective study was conducted. Data from 81,974 ARTI cases from seven medical institutions in Hangzhou City between January 2018 and December 2025 were collected. Cases with positive test results were analyzed for influenza A virus (FluA), influenza B virus (FluB), respiratory syncytial virus (RSV), adenovirus (ADV), *Mycoplasma pneumoniae* (MP), and *Chlamydia pneumoniae* (CP), and statistical analysis was performed according to the pandemic stage, age, sex, season, and co-infection status.

**Results:**

Among 81,974 patients, a total of 33,350 cases tested positive for pathogens, with an overall positive rate of 40.68%. MP (19.56%) and FluA (16.93%) were the most prevalent pathogens. The positivity rates of each pathogen showed statistically significant differences across the three stages before, during, and after the Coronavirus Disease 2019 (COVID-19) pandemic (all *P* < 0.001). Compared with the pre-COVID-19 pandemic period (2018–2019), the positive rates of FluA, FluB, and MP were significantly suppressed during the COVID-19 pandemic period (2020–2022), and then experienced a sharp “revenge rebound” in the post-COVID-19 pandemic period (2023–2025) (for example, the positive rate of MP rebounded from 5.43% to 21.09%). In contrast, the positive rates of RSV, ADV, and CP showed a persistent state of inhibition, but the absolute number of cases far exceeded that of before the COVID-19 pandemic. MP had the highest infection rate among 6– < 12 years old (38.31%), whereas FluA was more common in adults. The epidemiological distribution showed obvious seasonal heterogeneity: FluA/FluB/RSV were more prevalent in winter, MP was active in autumn, and ADV was more prevalent in summer. The co-infection rate was 7.22% (5,922 cases), and the most common combination was FluA + MP, accounting for 53.82% of all co-infections.

**Conclusion:**

From 2018 to 2025, the pathogen spectrum of ARTI in Hangzhou was mainly composed of MP and FluA. The COVID-19 pandemic and related non-pharmaceutical interventions (NPIs) have had a profound and heterogeneous impact on the epidemic pattern of respiratory pathogens. FluA, FluB, and MP showed the characteristics of “suppression-retaliatory rebound.” In the post-COVID-19 pandemic era, the activity of pathogens has clear seasonal, age, and gender distribution patterns, and the rate of mixed infections is high. The research results suggest that a dynamic monitoring system needs to be established, and targeted prevention and control measures should be implemented for key individuals and pathogens during high-incidence seasons.

## 1 Introduction

Acute Respiratory Tract Infection (ARTI) is a leading cause of outpatient visits and emergency department visits, hospitalizations, and substantial disease burden globally. The pathogen spectrum of ARTI is complex and encompasses viruses, bacteria, and atypical pathogens (1). In China, ARTI is a prevalent infectious disease, which can lead to severe clinical consequences, especially among children, the older adult(s), and individuals with underlying diseases (2). Clarifying the epidemic characteristics of pathogens in specific periods and regions holds crucial scientific value for guiding empirical clinical treatment, implementing precise public health intervention measures (such as vaccination and seasonal prevention), and establishing effective monitoring and early warning systems.

In recent years, the epidemic patterns of respiratory pathogens have changed. The global pandemic of Coronavirus Disease 2019 (COVID-19) that began at the end of 2019, along with the widespread implementation of Non-Pharmaceutical Interventions (NPIs) to contain its spread, such as wearing masks, maintaining social distancing, and restricting gatherings and travel, have profoundly altered the ecological and epidemiological characteristics of common respiratory pathogens (3). Multiple studies have confirmed that during the strict implementation of NPIs, the activity levels of many common respiratory viruses, such as influenza and respiratory syncytial virus (RSV), significantly decreased or even disappeared (4, 5). However, since the gradual adjustment and lifting of NPIs at the end of 2022, new trends have emerged in many parts of the world, including the resurgence of common respiratory pathogen activities, shift in epidemic seasons, and co-circulation or alternating prevalence of multiple pathogen (6). For example, post-COVID-19 pandemic surveillance in Guangdong Province revealed that the peak activity of influenza viruses has advanced, the proportion of MP infections has exceeded the pre-COVID-19 pandemic average, and there are significant age-related differences in pathogen distribution (7). These changes indicate that in the “post-COVID-19 pandemic era,” the original pathogen prevalence patterns based on historical data may no longer be applicable, and there is an urgent need for updates and analyses based on the latest monitoring data.

As an important central city and transportation hub in eastern China, Hangzhou has a dense population and high population mobility, making it an ideal sentinel site for monitoring the dynamics of respiratory infectious disease. However, there is currently a lack of systematic, long-term longitudinal research covering the complete cycle before the COVID-19 pandemic, during thepandemic, and after the implementation of “Category B Management for Category B Infectious Diseases” on the epidemiological characteristics of multiple pathogens among ARTI cases in Hangzhou, especially studies that simultaneously cover viruses and atypical pathogens. To address this gap, we conducted a multicenter retrospective study of 81,974 ARTI cases from seven hospitals in Hangzhou (2018–2025) to systematically characterize the epidemiological dynamics of six common respiratory pathogens before, during, and after the COVID-19 pandemic.

## 2 Materials and methods

### 2.1 Study population

This study aimed to systematically characterize the epidemiological features of six key respiratory pathogens—influenza A virus (FluA), influenza B virus (FluB), RSV, adenovirus (ADV), *Mycoplasma pneumoniae* (MP), and *Chlamydia pneumoniae* (CP)—among ARTI cases in Hangzhou, and to evaluate the long-term impact of the COVID-19 pandemic on local pathogen dynamics. A total of 81,974 patients from seven medical institutions in Hangzhou seven medical institutions in Hangzhou (see Supplementary Table 1 for details) between January 2018 and December 2025 were selected as study subjects. The diagnostic criteria for cases were as follows: pediatric cases were defined according to the Practical Pediatrics (Zhu Futang, 8th edition) (8), while adult cases were defined according to the Expert consensus on the diagnosis, treatment, and infection control of acute respiratory infections in adult outpatient and emergency departments (9), including: (1) Upper respiratory tract infection (URTI): presence of ≥2 symptoms—fever (body temperature ≥38 °C), cough, nasal congestion, rhinorrhea, sore throat, or sneezing—with a duration of ≤ 7 days. (2) Lower respiratory tract infection (LRTI): Clinical signs: increased respiratory rate, moist rales/whistling sounds in the lungs, or tracheal retractions; Radiological evidence (X-ray/CT): signs of pneumonia/bronchitis (e.g., patchy infiltrates, atelectasis). Exclusion criteria: Incomplete clinical data, incomplete pathogen detection results, or duplicate records. Clinical data from 1,431,696 individuals undergoing respiratory pathogen screening were collected in this study. After applying the inclusion and exclusion criteria, we excluded entries with incomplete pathogen detection, removed duplicate data from repeated hospitalizations and repeated outpatient visits (1,331,565 cases), as well as duplicate data between outpatient and inpatient records (18,157 cases). Ultimately, a total of 81,974 subjects were included in the statistical analysis (Figure 1).

**Figure 1:**
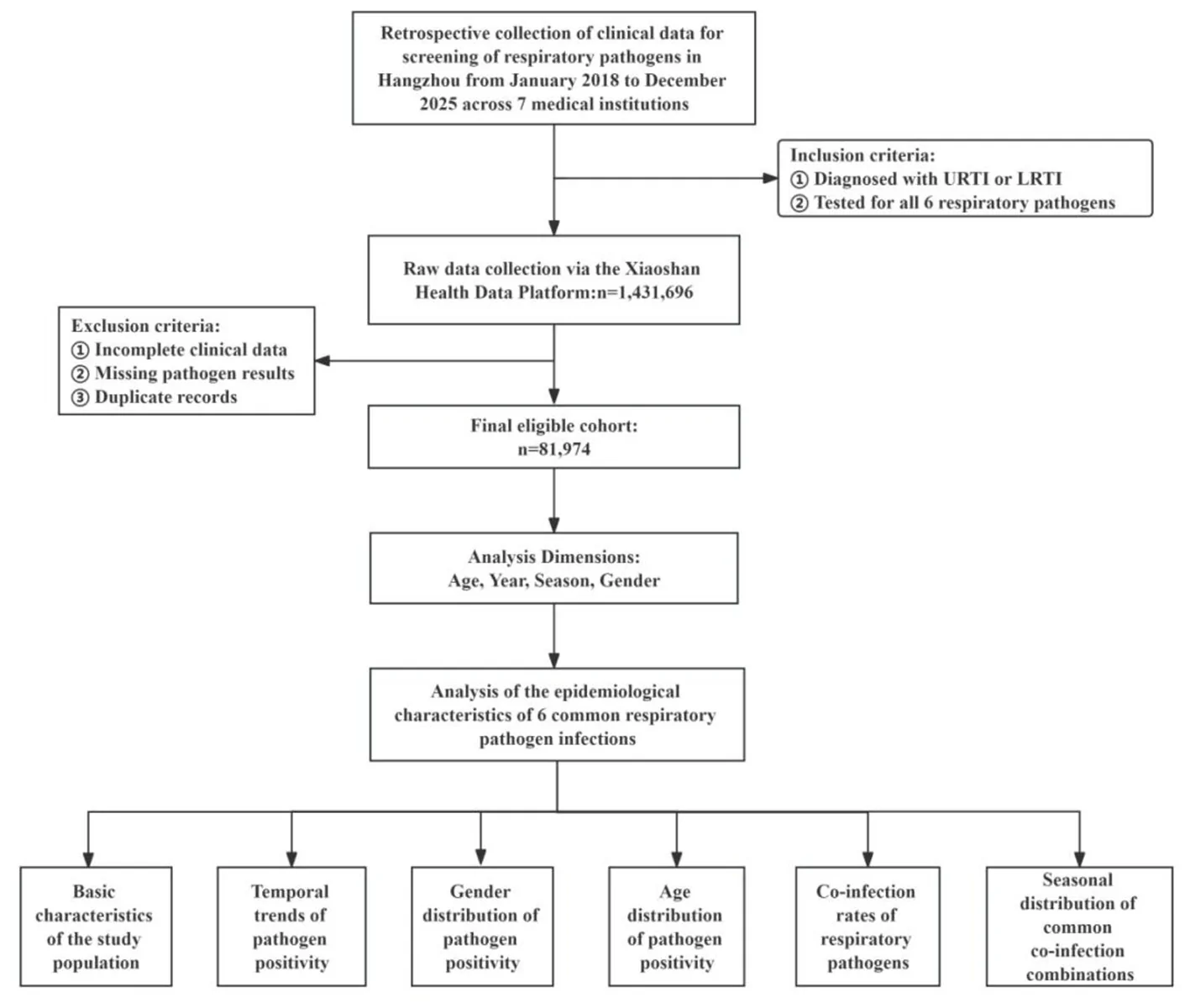
Flowchart of the study.

### 2.2 Data collection

Demographic and clinical data of enrolled cases were collected through the “Xiaoshan Health Data Platform” platform. The determination of infection status was based on any of the following positive test results: positive serum pathogen-specific IgM antibodies, or positive pathogen antigens or nucleic acids detected in respiratory tract samples. This study encompasses six common respiratory pathogens, including FluA, FluB, RSV, ADV, MP, and CP. For all six pathogens, specimen types included nasopharyngeal or oropharyngeal swabs, sputum, bronchoalveolar lavage fluid, and blood; detection methods included serological IgM assays, antigen detection, and nucleic acid amplification tests (NAAT), with the exception that antigen detection was not included for CP. Positivity criteria followed the manufacturers' instructions for each respective assay. Only patients with complete test results for all six target pathogens were included in the final analysis.

Grouping of study subjects: in accordance with the World Health Organization (WHO) criteria for age-stage division and population age stratification, the study subjects were divided into six age groups: 0– < 3 years (infant and toddler group), 3– < 6 years (preschool group), 6– < 12 years (school-age group), 12– < 18 years (adolescence group), 18– < 60 years (adult group), and ≥60 years (older adult(s) group). According to year span, the subjects were divided into three periods: 2018–2019, 2020–2022, and 2023–2025. Seasonal grouping included spring (March–May), summer (June–August), autumn (September–November), and winter (December–February of the following year). The chi-square (χ^2^) two-sided test was used for intergroup comparisons of qualitative data, and a *P*-value < 0.05 was considered statistically significant.

### 2.3 Statistical analysis

Data were exported to Excel, and statistical analyses were performed using R version 4.3.2. Normally distributed continuous data are presented as mean ± standard deviation, while non-normally distributed data are presented as median (interquartile range, IQR). Positivity rates for each study period were calculated as the total number of positive cases divided by the total number of tested cases across all years within that period. Count data are presented as frequencies and percentages. Comparisons between groups were performed using the chi-square test. All tests were two-sided, and the significance level was set at α = 0.05. Calculation formula: Positive rate (%) = (Number of positive cases detected among the screened population / Total number of screened cases) × 100%.

## 3 Results

### 3.1 Basic characteristics of the research subjects

Among 81,974 patients with ARTI, 33,350 were positive for pathogens, with a positivity rate of 40.68%. Specifically, the positive rate for outpatient cases was 44.64% (30,405/68,109), and that for inpatient cases was 19.83% (2,749/13,865). Among the 81,974 ARTI patients screened from 2018 to 2025, the number of cases each year was 1,915, 3,116, 1,086, 1,676, 2,963, 19,630, 31,639, and 19,949, respectively. The pathogen positive rates were 21.78% (417/1,915), 26.03% (811/3,116), 14.09% (153/1,086), 10.80% (181/1,676), 14.21% (421/2,963), 51.08% (10,027/19,630), 39.03% (12,350/31,639), and 44.08% (8,794/19,949), respectively, as shown in Figure 2. There were 41,996 males and 39,978 females, with a male-to-female ratio of 1.05:1. The median age was 24.00 (5.00, 37.00) years.

**Figure 2:**
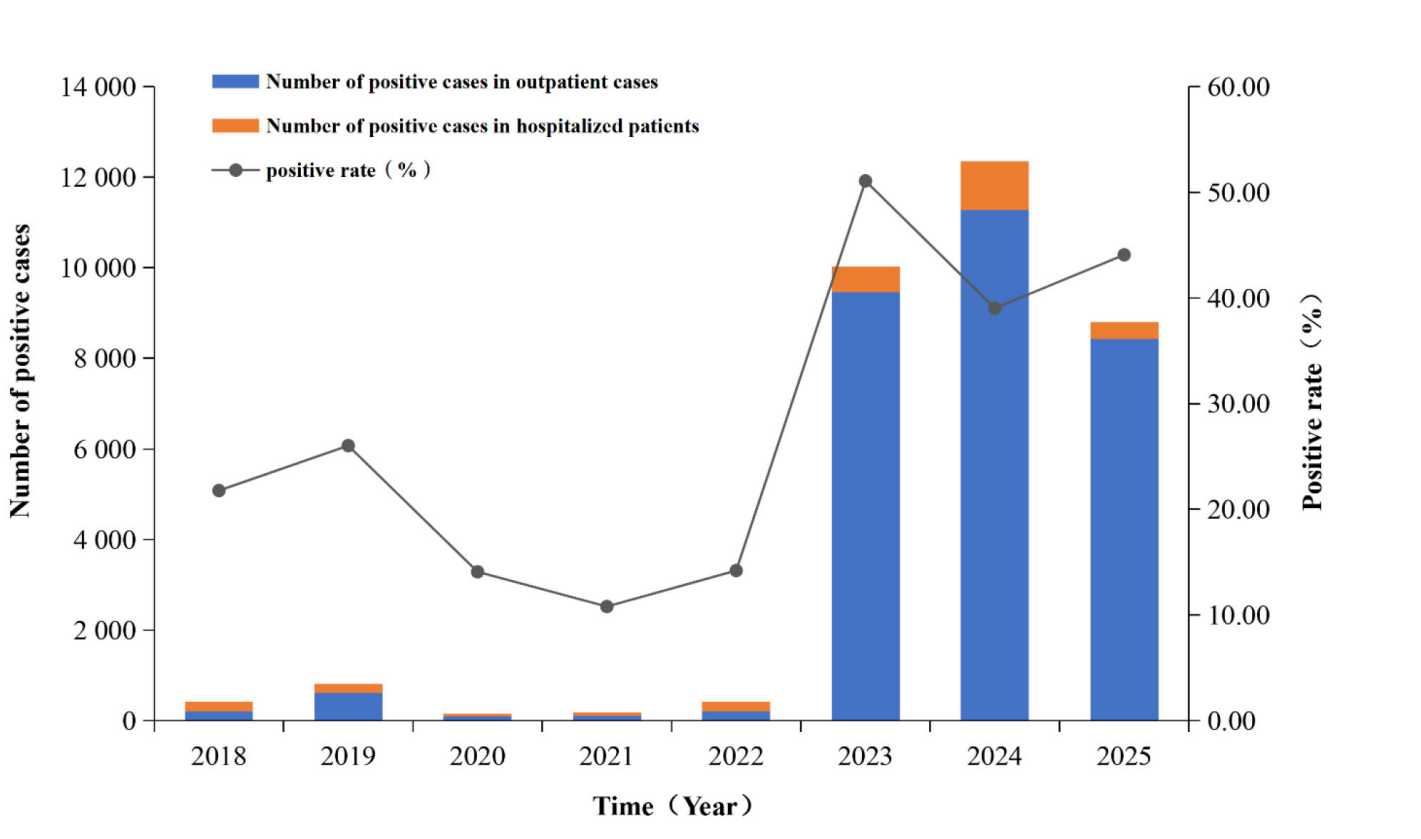
The number and positive rate of respiratory pathogens in acute respiratory infection cases in Hangzhou from 2018 to 2025.

Further stratified analysis revealed significant variations in the outpatient-inpatient positivity gap across subgroups (Supplementary Table 2). By age, the largest disparity was observed in children aged 3– < 6 years, with a positivity rate of 59.50% in outpatients compared to 22.44% in inpatients (difference of 36.9 percentage points). By time period, the gap widened markedly in the post-COVID-19 pandemic period (2023–2025: 51.27% vs. 22.93%) compared to the pre-COVID-19 pandemic period (2018–2019: 35.18% vs. 23.57%). By pathogen, Flu A, Flu B, and MP showed higher positivity in outpatients, whereas RSV, ADV, and CP were higher in inpatients.

### 3.2 Analysis of the positive detection status of pathogens and their temporal trends

From 2018 to 2025, among the acute respiratory infection cases in Hangzhou, the detection results of six common respiratory pathogens showed that MP had the highest overall positive rate (19.56%, 16,035/81,974), followed by FluA (16.93%, 13,876/81,974). The overall positivity rates of the remaining four pathogens ranged from 0.23% to 7.25%, including FluB (7.25%, 5,946/81,974), RSV (2.45%, 2,007/81,974), ADV (1.85%, 1,518/81,974), and CP (0.23%, 190/81,974).

Chi-square tests were performed to assess overall differences in the positive rates of each pathogen across the three period. The results indicated that the differences in the positive rates of each pathogen among the three periods were significant (all *P* < 0.001). Specifically, the positive rates of FluA, MP, and FluB showed a suppression during the COVID-19 pandemic period and rebounded significantly after the pandemic, presenting a typical “revenge-style epidemic” pattern, whereas, the positive rates of RSV, ADV, and CP showed a persistent state of inhibition, but the absolute number of cases was substantially higher than before the pandemic. The positive rates of MP and FluA from 2020 to 2022 demonstrated a decrease compared to 2018–2019 and from 2023 to 2025, showing an obvious “V-shaped” trend. Among them, the positive rate of MP during the COVID-19 pandemic period (5.43%) decreased by 61.1% compared with that before the pandemic (13.95%), and rebounded to 21.09% after the pandemic, an increase of 288.3% compared with the COVID-19 pandemic period; the positive rate of FluA during the COVID-19 pandemic period (1.57%) decreased by 58.5% compared with that before the pandemic (3.78%), and increased to 19.09% after the pandemic, an increase of 1115.9% compared with the COVID-19 pandemic period. The positive rates of ADV and CP from 2018 to 2019 were higher than those from 2020 to 2022 and from 2023 to 2025. The positive rate of ADV was 5.39% before the pandemic and decreased to 1.55% and 1.63% in the latter two periods, respectively; the positive rate of CP was 1.19% before the pandemic and decreased to 0.05% and 0.18% in the latter two periods, respectively. The positive rates of RSV were 3.08%, 2.90%, and 2.37% in the three periods, respectively, showing a gradual downward trend (*P* < 0.001). It is worth noting that although the overall positive rate decreased, the number of cases in the post-COVID-19 pandemic period (2023–2025) (1,686 cases) was much higher than that before the pandemic (155 cases), suggesting an increase in the absolute number of infected individuals (Table 1 and Figure 3).

**Table 1:** Comparison of positive rates of respiratory pathogen antibodies in different time periods.

Pathogens	2018–2019 year (*N* = 5,031)	2020–2022 year (*N* = 5,725)	2023–2025 year (*N* = 71,218)	χ^2^ value	*P*-value
Flu A	190 (3.78)	90 (1.57)	13,596 (19.09)	1815.708	< 0.001
Flu B	79 (1.57)	135 (2.36)	5,732 (8.05)	512.402	< 0.001
RSV	155 (3.08)	166 (2.90)	1,686 (2.37)	15.263	< 0.001
ADV	271 (5.39)	89 (1.55)	1,158 (1.63)	368.627	< 0.001
MP	702 (13.95)	311 (5.43)	15,022 (21.09)	933.074	< 0.001
CP	60 (1.19)	3 (0.05)	127 (0.18)	217.617	< 0.001

Data are presented as *N* (%). Positivity rates for each study period were calculated as the total number of positive cases divided by the total number of tested cases across all years within that period. χ^2^ values were derived from 3 (time periods) × 2 (positive/negative) contingency tables for each pathogen independently. Total positive rate was defined as the proportion of cases testing positive for at least one of the six target pathogens. FluA, influenza A virus; FluB, influenza B virus; RSV, respiratory syncytial virus; ADV, adenovirus; MP, *Mycoplasma pneumoniae*; CP, *Chlamydia pneumoniae*.

**Figure 3:**
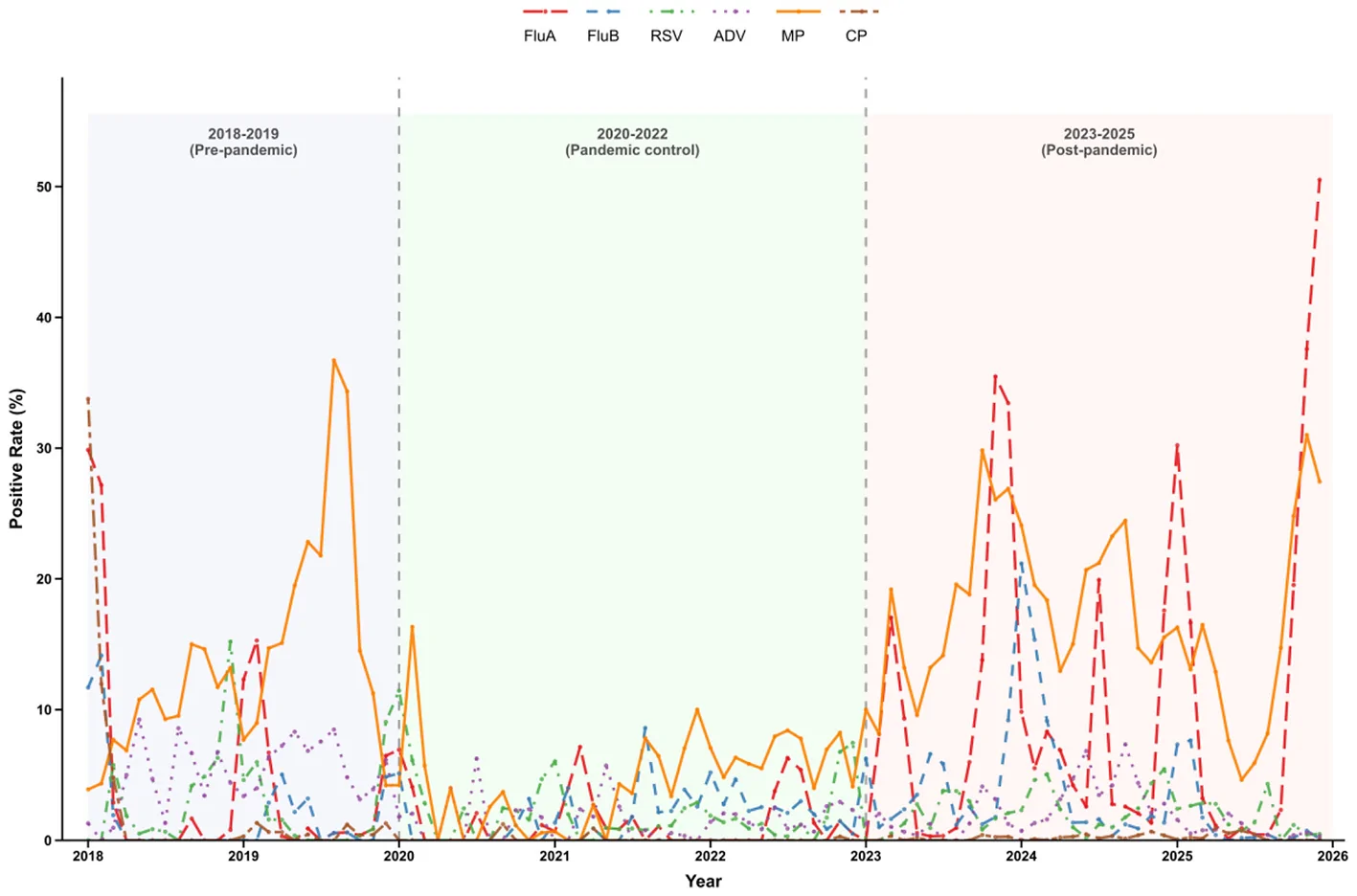
Monthly positive rate trends of six respiratory pathogens in Hangzhou, 2018–2025. FluA, influenza A virus; FluB, influenza B virus; RSV, respiratory syncytial virus; ADV, adenovirus; MP, *Mycoplasma pneumoniae*; CP, *Chlamydia pneumoniae*.

Upon further analysis of seasonal trends, the total positive detection rate of respiratory pathogens exhibited a fluctuating upward trend across seasons (χ^2^ = 3335.18, *P* < 0.001), indicating significant overall seasonal variation. However, pathogen-specific seasonal differences were not uniform: most pathogens showed statistically significant seasonal variation (*P* < 0.05), while CP exhibited no significant seasonal difference. Specifically, FluA, FluB, and RSV shared a winter-peaking pattern, with the highest positive rates in winter (21.90%, 10.87%, and 2.76%, respectively) and the lowest in summer or autumn. ADV peaked in summer (3.28%) and troughed in winter (1.19%), while MP peaked in autumn (21.62%) and troughed in spring (14.49%). CP showed consistently low positivity across all seasons (0.22%−0.26%), with no statistically significant differences. The overall pathogen detection rate is the highest in winter (48.43%) and the lowest in summer (24.90%). The distribution of respiratory pathogens in different seasons is detailed in Table 2.

**Table 2:** Distribution of respiratory pathogens in different seasons.

Pathogens	Spring (*N* = 15,061)	Summer (*N* = 7,803)	Autumn (*N* = 12,879)	Winter (*N* = 46,231)	χ^2^ value	*P*-value
Flu A	855 (5.68)	360 (4.61)	2,538 (19.71)	10,123 (21.90)	3079.60	< 0.001
Flu B	627 (4.16)	123 (1.58)	171 (1.33)	5,025 (10.87)	2158.36	< 0.001
RSV	399 (2.65)	87 (1.11)	244 (1.89)	1,277 (2.76)	96.24	< 0.001
ADV	354 (2.35)	256 (3.28)	358 (2.78)	550 (1.19)	280.80	< 0.001
MP	2,182 (14.49)	1,228 (15.74)	2,785 (21.62)	9,840 (21.28)	440.97	< 0.001
CP	39 (0.26)	17 (0.22)	29 (0.23)	105 (0.23)	0.61	0.893
Total	3,890 (25.83)	1,943 (24.90)	5,129 (39.82)	22,388 (48.43)	3335.18	< 0.001

Data are presented as *N* (%). χ^2^ values were derived from 4 (seasons) × 2 (positive/negative) contingency tables for each pathogen independently. Total positive rate was defined as the proportion of cases testing positive for at least one of the six target pathogens. FluA, influenza A virus; FluB, influenza B virus; RSV, respiratory syncytial virus; ADV, adenovirus; MP, *Mycoplasma pneumoniae*; CP, *Chlamydia pneumoniae*.

### 3.3 Analysis of the positive detection of pathogens and their distribution by gender

Among the 81,974 patients with respiratory tract infections included in the study, male patients accounted for 48.77% (39,978/81,974), and female patients accounted for 51.23% (41,996/81,974). The overall positive detection rate of respiratory pathogens in male patients was 39.52% (15,798/39,978), and that in female patients was 41.79% (17,552/41,996). Comparisons of positive rates between genders were performed separately for each pathogen and for the overall positivity using chi-square tests. The difference between the two groups was statistically significant (χ^2^ = 43.937, *P* < 0.001). Comparison of the positive detection rates of respiratory pathogens between different genders showed that the detection rate of ADV in male patients was higher than that in female patients (2.03% vs. 1.68%, χ^2^ = 13.233, *P* < 0.001), the detection rate of MP in female patients was higher than that in male patients (21.50% vs. 17.52%, χ^2^ = 205.924, *P* < 0.001), and the detection rate of RSV in female patients was slightly higher than that in male patients (2.56% vs. 2.34%, χ^2^ = 4.011, *P* = 0.045). There was no statistically significant difference in the detection rates of FluA virus and CP between different genders (*P* > 0.05) (Table 3).

**Table 3:** Comparison of positive detection rates of respiratory pathogens by gender.

Pathogens	Male (*N* = 39,978)	Female (*N* = 41,996)	χ^2^ value	*P*-value
Flu A	6,810 (17.03)	7,066 (16.83)	0.621	0.431
Flu B	2,890 (7.23)	3,056 (7.28)	0.063	0.802
RSV	934 (2.34)	1,073 (2.56)	4.011	0.045
ADV	811 (2.03)	707 (1.68)	13.233	< 0.001
MP	7,005 (17.52)	9,030 (21.50)	205.924	< 0.001
CP	90 (0.23)	100 (0.24)	0.099	0.753
Total	15,798 (39.52)	17,552 (41.79)	43.937	< 0.001

Data are presented as *N* (%). χ^2^ values were derived from 2 (genders) × 2 (positive/negative) contingency tables for each pathogen independently. Total positive rate was defined as the proportion of cases testing positive for at least one of the six target pathogens. FluA, influenza A virus; FluB, influenza B virus; RSV, respiratory syncytial virus; ADV, adenovirus; MP, *Mycoplasma pneumoniae*; CP, *Chlamydia pneumoniae*.

### 3.4 Analysis of the positive detection status of pathogens and their age distribution

When comparing the overall positive detection rates of respiratory pathogens among different age groups, the differences were statistically significant (χ^2^ = 2970.17, *P* < 0.001). The total positive detection rates were the highest in the 6– < 12 years old and the 12– < 18 years old, reaching 54.95% and 51.55%, respectively, while the positive detection rate was the lowest in the < 3 years group (26.95%). MP was the predominant pathogen in younger age groups, with the highest positive rates in the 0– < 3 years (14.50%), 3– < 6 years (24.97%), 6– < 12 years (38.31%), and 12– < 18 years (27.03%) groups, while FluA dominated in adults aged 18– < 60 years (20.92%) and the older adult(s) aged ≥60 years (14.61%). When comparing the positive detection rates of different respiratory pathogens among different age groups, all the differences were statistically significant (*P* < 0.05), as shown in Table 4.

**Table 4:** Distribution of respiratory pathogens in different age groups.

Pathogens	< 3 years (*N* = 11,179)	3– < 6 years (*N* = 11,402)	6– < 12 years (*N* = 14,348)	12– < 18 years (*N* = 6,900)	18– < 60 years (*N* = 28,841)	≥60 years (*N* = 9,304)	χ^2^ value	*P*-value
Flu A	671 (6.00)	1,371 (12.02)	2,754 (19.19)	1,687 (24.45)	6,034 (20.92)	1,359 (14.61)	1836.71	< 0.001
Flu B	231 (2.07)	456 (4.00)	1,066 (7.43)	596 (8.64)	2,945 (10.21)	652 (7.01)	1022.76	< 0.001
RSV	592 (5.30)	302 (2.65)	117 (0.82)	57 (0.83)	537 (1.86)	402 (4.32)	795.68	< 0.001
ADV	272 (2.43)	369 (3.24)	286 (1.99)	83 (1.20)	377 (1.31)	131 (1.41)	215.75	< 0.001
MP	1,621 (14.5)	2,847 (24.97)	5,497 (38.31)	1,865 (27.03)	3,639 (12.62)	566 (6.08)	5802.38	< 0.001
CP	23 (0.21)	30 (0.26)	28 (0.2)	28 (0.41)	56 (0.19)	25 (0.27)	12.99	0.023
Total	3,013 (26.95)	4,472 (39.22)	7,884 (54.95)	3,557 (51.55)	11,739 (40.70)	2,685 (28.86)	2970.17	< 0.001

Data are presented as *N* (%). χ^2^ values were derived from 6 (age groups) × 2 (positive/negative) contingency tables for each pathogen independently. Total positive rate was defined as the proportion of cases testing positive for at least one of the six target pathogens. FluA, influenza A virus; FluB, influenza B virus; RSV, respiratory syncytial virus; ADV, adenovirus; MP, *Mycoplasma pneumoniae*; CP, *Chlamydia pneumoniae*.

### 3.5 Analysis of mixed infections of pathogens

The overall detection rate of respiratory pathogens in inpatients was 40.68% (33,350/81,974), among which the rate of single infection was 33.46% (27,428/81,974) and that of multiple infection was 7.22% (5,922/81,974). Multiple infections (≥2 pathogens) included double infection at 6.89% (5,650/81,974), triple infection at 0.30% (249/81,974), and quadruple infection at 0.02% (19/81,974). Chi-square tests were performed for each infection type by comparing the number of positive cases against the number of negative cases between subgroups, using R × C contingency tables. The overall infection rate, single-infection rate, double-infection rate, and multiple-infection detection rate in female patients were significantly higher than those in male patients (*P* < 0.05). There were statistically significant differences in the overall detection rate of respiratory pathogens and the detection rates of single infection, double infection, triple infection, and multiple infection among different seasons (*P* < 0.001). The infection detection rate in summer (24.90%) was lower than those in winter (48.43%), autumn (39.82%), and spring (25.83%). There were also statistically significant differences in the overall detection rate of respiratory pathogens and the detection rates of single infection, double infection, triple infection, and multiple infection among different age groups (*P* < 0.001). The highest detection rate was generally in the 6– < 12 years old (54.95%), as shown in Table 5.

**Table 5:** Patterns of respiratory pathogen co-infections stratified by gender, season, and age.

Variable	Group	*N*	Single infection	Co-infection	Triple infection	Quadruple infection	Multiple infection
Gender	Male	39,978	13,191 (33.00)	2,486 (6.22)	110 (0.28)	8 (0.02)	2,607 (6.52)
	Female	41,996	14,237 (33.90)	3,164 (7.53)	139 (0.33)	11 (0.03)	3,315 (7.89)
	χ^2^ value		7.497	55.036	1.928	0.124	57.361
	*P*-value		0.006	< 0.001	0.165	0.725	< 0.001
Season	Winter	46,231	18,060 (39.06)	4,145 (8.97)	166 (0.36)	14 (0.03)	4,328 (9.36)
	Summer	7,803	1,822 (23.35)	114 (1.46)	7 (0.09)	0 (0.00)	121 (1.55)
	Spring	15,061	3,361 (22.32)	493 (3.27)	35 (0.23)	1 (0.01)	529 (3.51)
	Autumn	12,879	4,185 (32.49)	898 (6.97)	41 (0.32)	4 (0.03)	944 (7.33)
	χ^2^ value		1856.009	975.93	19.099	–	999.708
	*P*-value		< 0.001	< 0.001	< 0.001	0.195^a^	< 0.001
Age	0~ < 3 years	11,179	2,629 (23.52)	372 (3.33)	11 (0.10)	1 (0.01)	384 (3.44)
	3~ < 6 years	11,402	3,610 (31.66)	821 (7.20)	41 (0.36)	0 (0.00)	862 (7.56)
	6~ < 12 years	14,348	6,072 (42.32)	1,760 (12.27)	52 (0.36)	0 (0.00)	1,812 (12.63)
	12~ < 18 years	6,900	2,816 (40.81)	724 (10.49)	16 (0.23)	1 (0.01)	741 (10.74)
	18~ < 60 years	28,841	10,005 (34.69)	1,631 (5.66)	91 (0.32)	12 (0.04)	1,734 (6.01)
	≥60 years	9,304	2,296 (24.68)	342 (3.68)	38 (0.41)	5 (0.05)	389 (4.18)
	χ^2^ value		1528.2	1,226.946	23.047	**–**	1185.688
	*P*-value		< 0.001	< 0.001	< 0.001	0.006^a^	< 0.001
Total		81,974	27,428 (33.46)	5,650 (6.89)	249 (0.30)	19 (0.02)	5,922 (7.22)

Data are presented as *N* (%). Total positive rate was defined as the proportion of cases testing positive for at least one of the six target pathogens. ^a^Use Fisher's exact test.

### 3.6 Seasonal distribution of common multiple infection combinations

In total, 5,922 cases of multiple infections with various pathogen combinations were identified; the six most prevalent co-infection combinations across all seasons are shown in Figure 4. The most common co-infection combination was FluA + MP, with 3187 cases (53.82%); followed by FluB + MP, with 1,191 cases (20.11%); and the third most common was the FluA + FluB combination, with 328 cases (5.54%). Other relatively common combinations included MP + ADV (3.87%), MP + RSV (3.16%), etc. For the remaining combinations, please refer to Supplementary Table 3. The distribution of co-infection combinations exhibited distinct seasonal patterns (Figure 4). FluA + MP was the predominant combination in both autumn (5.36%) and winter (5.10%). In spring, FluA + FluB had the highest detection rate (1.06%), followed by FluA + MP (0.79%). In contrast, ADV + MP was the most frequently detected combination in summer (0.63%), although overall co-infection rates remained low during this season. Additionally, FluB + MP showed a notable prevalence in winter (2.38%), ranking as the second most common co-infection combination during this season.

**Figure 4:**
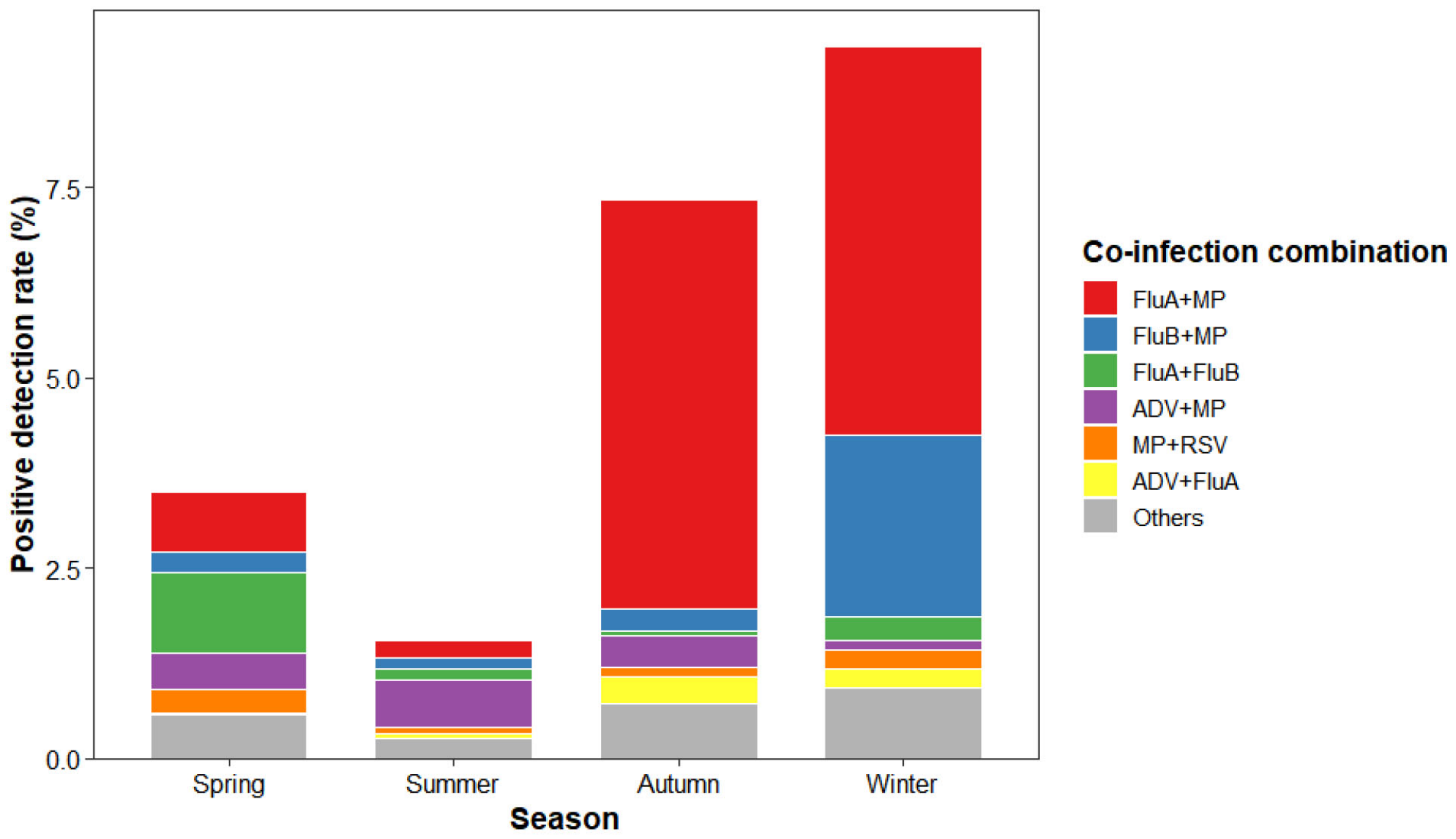
Seasonal distribution of the six most prevalent co-infection combinations. FluA, influenza A virus; FluB, influenza B virus; RSV, respiratory syncytial virus; ADV, adenovirus; MP, *Mycoplasma pneumoniae*; CP, *Chlamydia pneumoniae*.

## 4 Discussion

This study conducts a multi-center retrospective analysis of a total of 81,974 ARTI cases in Hangzhou from 2018 to 2025, describing the temporal variations in the epidemic patterns of six major respiratory pathogens before, during, and after the COVID-19 pandemic. Our data confirm the rebound phenomenon of pathogen activity observed globally in the post-COVID-19 pandemic era. However, the recovery patterns varied markedly across pathogens: Flu A and MP rebounded rapidly to pre- COVID-19 pandemic levels, whereas RSV and ADV exhibited delayed or incomplete recovery. These divergent trajectories resulted in a reshaped epidemiological landscape, characterized by altered seasonal peaks and shifts in the age groups predominantly affected. These findings have important implications for understanding the complexity of the “immunity debt” hypothesis, optimizing clinical practice, and reconstructing the local surveillance and early-warning system.

Research findings indicate that there are significant differences in the inhibitory effects of NPIs on common respiratory pathogens. The positive rates of Influenza A (FluA), Influenza B (FluB), and MP were significantly suppressed during the period of epidemic prevention and control from 2020 to 2022, and then showed a sharp “revenge rebound” from 2023 to 2025. In particular, the positive rate of FluA in the post-COVID-19 pandemic period (19.09%) was more than five times that before the COVID-19 pandemic (3.78%), representing a rebound increase of over 11-fold compared with the COVID-19 pandemic period (1.57%). This phenomenon is consistent with reports from many countries around the world and is widely attributed to the “immunity debt,” that is, NPIs led to a decrease in natural infections among the population and an accumulation of susceptible individuals. Once the interventions were lifted, the pathogens spread rapidly among the population with immunity gaps (7, 10, 11).

However, the decline in the positive rates of ADV, CP, and RSV in this study challenges the view that the single “immune debt” hypothesis can universally explain the dynamics of all pathogens, and reveals the “asymmetry” of the impact of NPIs (12). This disparity is the result of the combined effects of pathogen characteristics, population immunodynamics, and persistent changes in behavioral patterns after the pandemic. Taking ADV as an example, it has multiple transmission routes such as droplet, fecal-oral, and contact (13), this niche breadth may have buffered the impact of NPIs, preventing the complete interruption of its transmission during the pandemic and thus avoiding the accumulation of a large susceptible population like that of influenza viruses and MP (14). The latter two are highly dependent on droplet transmission and are strongly suppressed under NPIs, which in turn leads to a “revenge epidemic” after the lifting of the interventions. It is worth noting that the interpretation of the “decline in the positive rate” requires extra caution. Although the positive rates of ADV, CP, and RSV show a persistent state of inhibition, the absolute number of detected cases during the post-COVID-19 pandemic period (2023–2025) far exceeds the pre-COVID-19 pandemic level (15–17). This strongly suggests that the simple positive rate may underestimate the actual intensity of community transmission and disease burden of this pathogen in the post-COVID-19 pandemic stage. The reasons for this phenomenon may include: the recovery and increase in the overall number of medical consultations after the pandemic, the improvement in the willingness to undergo testing, and the sample dilution effect caused by the co-circulation with other pathogens (such as MP, FluA) (18, 19). Therefore, our data indicate that the evolution of respiratory pathogens in the “post-pandemic era” is not simply a “return to the baseline,” but rather a profound “restructuring of ecological niches.” With their high transmission efficiency, MP and influenza viruses have significantly increased their epidemiological weight in the context of expanded susceptible pools and the restoration of transmission conditions; while other pathogens have their activity levels reshaped either due to their transmission characteristics or because of sustained behavioral suppression (20). This suggests that in the surveillance during the “post-COVID-19 pandemic era,” it is necessary to combine multi-dimensional indicators such as the positive rate and the absolute number of cases, and take into account confounding factors such as changes in medical seeking behavior to more accurately assess the real epidemic situation and disease burden of various pathogens.

The stratified outpatient-inpatient comparison further illustrates how healthcare-seeking behavior shapes surveillance data. The universal lower inpatient positivity rate across age groups likely reflects that inpatients include more non-infectious admissions and prior antibiotic use. The widening gap post- COVID-19 pandemic suggests a surge in mild community infections seeking outpatient care. Notably, RSV and ADV were higher in inpatients, consistent with their tendency to cause more severe disease requiring hospitalization (21, 22).

This study clearly identifies that MP has become the pathogen with the highest positive rate (19.56%) among ARTI cases in the Hangzhou area, far exceeding that of FluA (16.93%). Age distribution analysis showed that MP infection peaked in children aged 6– < 12 years (38.31%), consistent with recent reports from multiple domestic regions (23, 24). The higher overall positivity of MP compared to RSV in our cohort reflects their distinct age-specific ecological niches rather than diminished clinical relevance of RSV. Age-stratified analysis (Table 4) revealed that MP positivity increased markedly with age, peaking in school-aged children (6– < 12 years: 38.31%), whereas RSV activity was concentrated in infants and toddlers (< 3 years: 5.30%). In older age groups, MP positivity far exceeded RSV (e.g., 6– < 12 years: 38.31% vs. 0.82%), which explains why MP appears more prevalent in the all-ages cohort. RSV remains a critical pathogen causing severe lower respiratory tract infections predominantly in young children, and its importance should not be underestimated despite the lower overall positivity rate in this study. The high susceptibility of 6– < 12 years old can be explained by aspects such as their group living characteristics, immune system development features, and social behavior traits. As semi-enclosed and high-density environments, schools provide ideal conditions for the transmission of MP through droplets. Meanwhile, since children's immune systems are not fully developed and their specific immune memory of MP is relatively limited, these factors collectively make this population the primary victims of MP infection (25).

In addition, research has found that the mixed infection pattern (accounting for 17.8% of the total positive cases) provides important clues for understanding the severity of the disease. FluA + MP is the most predominant co-infection combination (accounting for 53.82% of all multiple infections), and this phenomenon deserves high-level attention. Basic research suggests that influenza virus infection can damage respiratory epithelial cells and disrupt the function of the mucosal barrier, thereby creating favorable conditions for the secondary colonization and infection of pathogens such as MP (26). Clinically, co-infection of influenza and mycoplasma may be associated with a longer fever duration, a higher risk of pulmonary imaging progression, and more complex treatment decisions (27). Our data indicate that such co-infections are more prevalent in winter, coinciding with the influenza season. This suggests that during the influenza season, for patients diagnosed with influenza, especially child patients, the possibility of MP co-infection should be vigilant to avoid missed diagnosis and delayed treatment. This supports the clinical value of conducting multiplex pathogen detection, including influenza virus and MP, for severe or poorly-responding ARTI cases during the influenza epidemic season. Mixed infections may increase the severity of diseases and the complexity of treatment (28, 29). A study on lower respiratory tract infections in children has indicated that viral-bacterial co-infection is associated with a higher hospitalization rate and a longer length of hospital stay (30). The high incidence of co-infection in this study suggests that clinicians should consider conducting multiplex pathogen testing when dealing with severe or complex ARTI cases to guide the rational use of antimicrobial agents.

The strength of this study lies in the fact that it is the first to construct a prevalence baseline for respiratory pathogens covering the entire cycle of the pandemic based on multi-center and large-sample clinical data from Hangzhou. The extremely long monitoring time span (8 years), which covers the pre-COVID-19 pandemic, COVID-19 pandemic, and post-COVID-19 pandemic period, along with a large sample size (>80,000 cases), provides a rare long-term dynamic panorama of multiple pathogens. However, this study also has limitations. Firstly, as a retrospective study, it relies on routine medical records, and the detection methods, specimen types, and platforms varied across institutions and periods, though all patients were tested for all six pathogens, potentially affecting positivity accuracy. Secondly, other important pathogens such as SARS-CoV-2 and rhinovirus are not included, resulting in an incomplete pathogen spectrum. Additionally, during data extraction, only the aggregate diagnosis of ARTI was collected for each case, and the specific diagnosis (URTI vs. LRTI) was not individually documented, making it difficult to perform stratified analysis by infection site. Future research should adopt a prospective design that employs a unified multiplex molecular detection platform, systematically records clinical diagnoses (including URTI vs. LRTI), expands the pathogen panel to cover emerging viruses, and incorporates community-based sampling to minimize selection bias.

In summary, MP and FluA have become the dominant respiratory pathogens in Hangzhou post-COVID-19 pandemic, peaking in 6– < 12 years old and winter, with FluA + MP as the key co-infection pattern requiring heightened vigilance. These findings call for updating empirical treatment and prevention strategies, including incorporating MP into routine pediatric surveillance, strengthening influenza vaccination, and maintaining continuous multi-pathogen sentinel surveillance.

## Data Availability

The raw data supporting the conclusions of this article will be made available by the authors, without undue reservation.
