# Supplementary material for: Shifts in acute respiratory pathogen epidemiology: an 8-year surveillance study in Hangzhou, China (2018–2025)
Source: Front Public Health. 2026 Sep 30;14:1907370. doi: 10.3389/fpubh.2026.1907370 (PMC13663657; doi:10.3389/fpubh.2026.1907370)
Supplement: Supplementary file 1 [file Supplementary_File_1.docx]

**Supplementary Table 1.** Details of the seven participating medical institutions in Hangzhou

| **No.** | **Institution** | **Type** | **Grade** | **Location** |
| --- | --- | --- | --- | --- |
| 1 | Zhejiang Xiaoshan Hospital | General hospital | Tertiary, Grade B | Beigan Subdistrict |
| 2 | The First People's Hospital of Xiaoshan District, Hangzhou City | General hospital | Tertiary, Grade A | Chengxiang Subdistrict |
| 3 | The Second People's Hospital of Xiaoshan District, Hangzhou City | General hospital | Secondary, Grade B | Guali Town |
| 4 | The Third People's Hospital of Xiaoshan District, Hangzhou City | General hospital | Secondary, Grade A | Linpu Town |
| 5 | Suoshan Subdistrict Community Health Center | Primary care | Primary, Grade A | Suoshan Subdistrict |
| 6 | Jingjiang Subdistrict Community Health Center | Primary care | Primary | Jingjiang Subdistrict |
| 7 | Xinjie Subdistrict Community Health Center | Primary care | Primary, Grade A | Xinjie Subdistrict |

**Supplementary Table 2.** Prevalence of Respiratory Pathogens in Outpatient and Inpatient Settings Across Different Stratification Variables

| Stratification variable | Level | Total N | Outpatient N | Inpatient N | Outpatient positive (rate%) | Inpatient positive (rate%) | χ² | P value |
| --- | --- | --- | --- | --- | --- | --- | --- | --- |
| Age group | 0~<3 years | 11179 | 2784 | 8395 | 1443 (51.83%) | 1570 (18.7%) | 1163.88 | <0.001 |
|  | 3~<6 years | 11402 | 5163 | 6239 | 3072 (59.5%) | 1400 (22.44%) | 1626.21 | <0.001 |
|  | 6~<12 years | 14348 | 9922 | 4426 | 6474 (65.25%) | 1410 (31.86%) | 1377.23 | <0.001 |
|  | 12~<18 years | 6900 | 6276 | 624 | 3416 (54.43%) | 141 (22.6%) | 229.01 | <0.001 |
|  | 18~<60 years | 28841 | 24697 | 4144 | 11071 (44.83%) | 668 (16.12%) | 1210.50 | <0.001 |
|  | ≥60 years | 9304 | 5044 | 4260 | 1943 (38.52%) | 742 (17.42%) | 499.94 | <0.001 |
| Time period | 2018–2019 | 5031 | 742 | 4289 | 261 (35.18%) | 1011 (23.57%) | 44.47 | <0.001 |
|  | 2020–2022 | 5725 | 286 | 5439 | 58 (20.28%) | 710 (13.05%) | 11.60 | <0.001 |
|  | 2023–2025 | 71218 | 52858 | 18360 | 27100 (51.27%) | 4210 (22.93%) | 4441.11 | <0.001 |
| Gender | Female | 39978 | 25154 | 14824 | 12658 (50.32%) | 3140 (21.18%) | 3312.52 | <0.001 |
|  | Male | 41996 | 28732 | 13264 | 14761 (51.37%) | 2791 (21.04%) | 3430.98 | <0.001 |
| Pathogen | Flu A | 81974 | 53886 | 28088 | 13018 (24.16%) | 858 (3.05%) | 5846.30 | <0.001 |
|  | Flu B | 81974 | 53886 | 28088 | 5266 (9.77%) | 680 (2.42%) | 1482.21 | <0.001 |
|  | RSV | 81974 | 53886 | 28088 | 1104 (2.05%) | 903 (3.21%) | 104.64 | <0.001 |
|  | ADV | 81974 | 53886 | 28088 | 872 (1.62%) | 646 (2.3%) | 46.83 | <0.001 |
|  | MP | 81974 | 53886 | 28088 | 12887 (23.92%) | 3148 (11.21%) | 1894.12 | <0.001 |
|  | CP | 81974 | 53886 | 28088 | 60 (0.11%) | 130 (0.46%) | 97.13 | <0.001 |

Note: Abbreviations: FluA, influenza A virus; FluB, influenza B virus; RSV, respiratory syncytial virus; ADV, adenovirus; MP, Mycoplasma pneumoniae; CP, Chlamydia pneumoniae.

**Supplementary Table 3.** Distribution of Pathogen Co-infection Combinations.

| **Co-infection combination** | **The quantity of pathogens** | **Cases** | **proportion（%）** |
| --- | --- | --- | --- |
| FLU A+MP | 2 | 3187 | 53.82 |
| FLU B+MP | 2 | 1191 | 20.11 |
| FLU A+FLU B | 2 | 328 | 5.54 |
| MP+ADV | 2 | 229 | 3.87 |
| MP+RSV | 2 | 187 | 3.16 |
| FLU A+ADV | 2 | 164 | 2.77 |
| FLU A+RSV | 2 | 124 | 2.09 |
| FLU B+RSV | 2 | 80 | 1.35 |
| FLU A+FLU B+MP | 3 | 63 | 1.06 |
| RSV+ADV | 2 | 61 | 1.03 |
| FLU B+ADV | 2 | 41 | 0.69 |
| FLU A+MP+RSV | 3 | 35 | 0.59 |
| FLU A+MP+ADV | 3 | 32 | 0.54 |
| FLU A+CP | 2 | 25 | 0.42 |
| CP+MP | 2 | 24 | 0.41 |
| FLU A+RSV+ADV | 3 | 22 | 0.37 |
| FLU A+FLU B+RSV | 3 | 19 | 0.32 |
| MP+RSV+ADV | 3 | 19 | 0.32 |
| FLU B+MP+RSV | 3 | 15 | 0.25 |
| FLU A+CP+MP | 3 | 11 | 0.19 |
| FLU B+MP+ADV | 3 | 11 | 0.19 |
| FLU B+RSV+ADV | 3 | 9 | 0.15 |
| FLU B+CP+MP | 3 | 5 | 0.08 |
| FLU A+FLU B+MP+RSV | 4 | 4 | 0.07 |
| CP+ADV | 2 | 4 | 0.07 |
| FLU A+FLU B+MP+ADV | 4 | 3 | 0.05 |
| FLU A+FLU B+CP | 3 | 3 | 0.05 |
| FLU A+FLU B+CP+MP+RSV | 5 | 3 | 0.05 |
| FLU A+MP+RSV+ADV | 4 | 3 | 0.05 |
| FLU A+CP+MP+ADV | 4 | 3 | 0.05 |
| FLU B+MP+RSV+ADV | 4 | 3 | 0.05 |
| FLU B+CP | 2 | 3 | 0.05 |
| CP+MP+RSV | 3 | 3 | 0.05 |
| CP+RSV | 2 | 2 | 0.03 |
| FLU A+FLU B+RSV+ADV | 4 | 1 | 0.02 |
| FLU A+FLU B+CP+MP+RSV+ADV | 6 | 1 | 0.02 |
| FLU A+FLU B+ADV | 3 | 1 | 0.02 |
| FLU A+CP+RSV | 3 | 1 | 0.02 |
| FLU B+CP+MP+RSV | 4 | 1 | 0.02 |
| FLU B+CP+MP+ADV | 4 | 1 | 0.02 |

Note: Abbreviations: FluA, influenza A virus; FluB, influenza B virus; RSV, respiratory syncytial virus; ADV, adenovirus; MP, Mycoplasma pneumoniae; CP, Chlamydia pneumoniae.
